# Supplementary material for: Structure and Function of the T4 Spackle Protein Gp61.3
Source: Viruses. 2020 Sep 24;12(10):1070. doi: 10.3390/v12101070 (PMC7650644; doi:10.3390/v12101070)
Supplement: Supplementary file 1 [file viruses-12-01070-s001.zip › viruses-918776ForXML.docx]

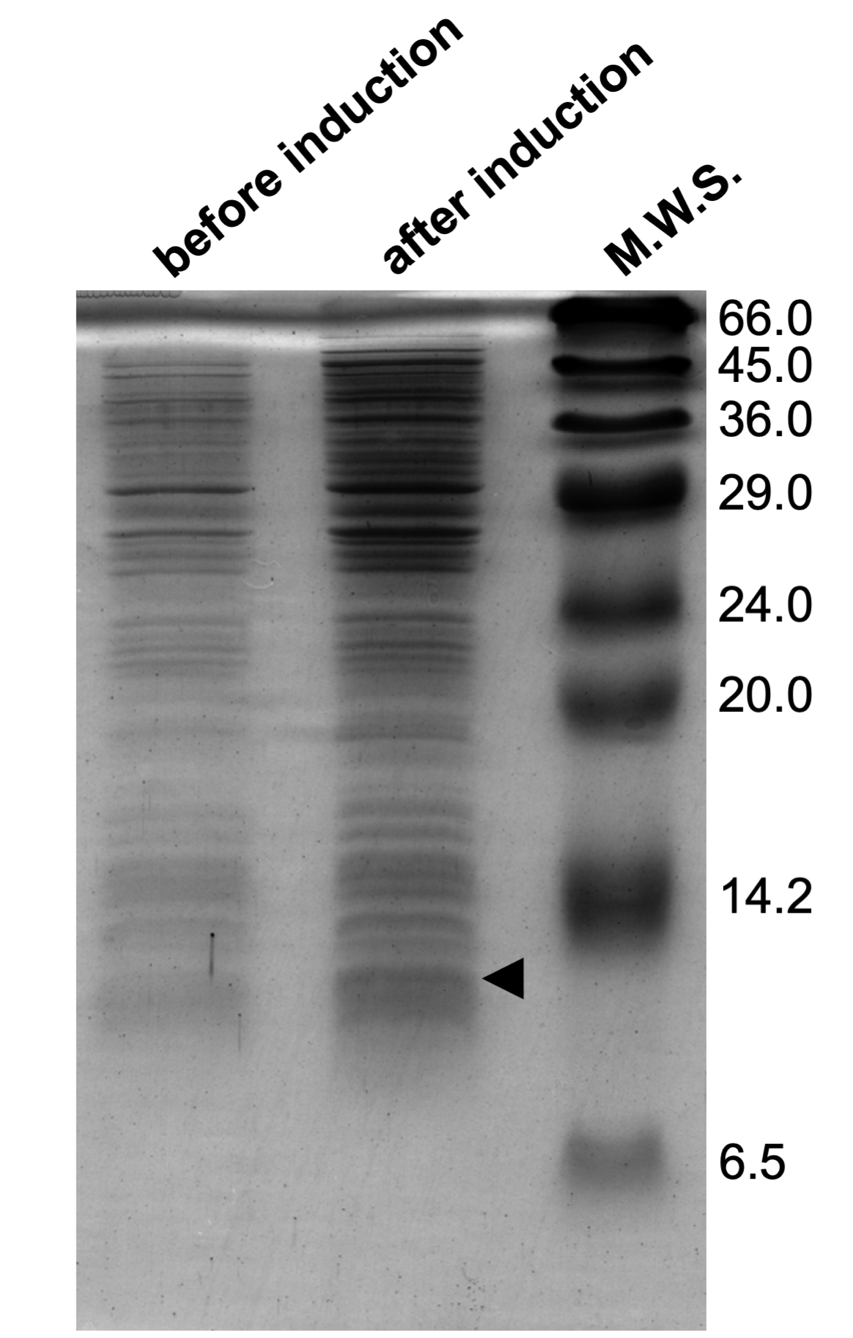


**Figure S1.** Expression of gp61.3 is visualized on a 17% SDS-PAGE. The arrowhead indicates the band corresponding to gp61.3 in the total cell lysate.


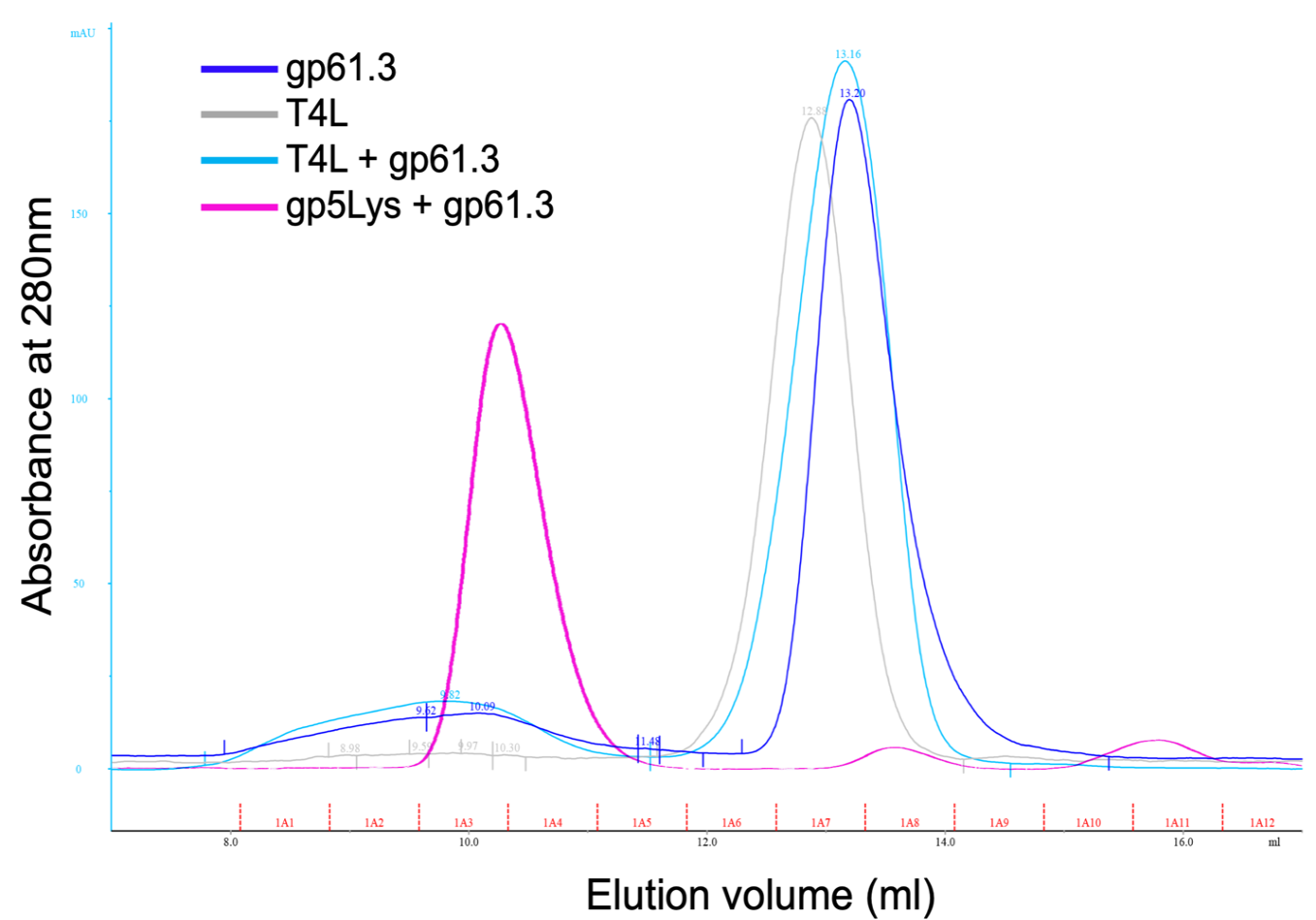


**Figure S2.** T4L does not form a complex with gp61.3. Size exclusion chromatograms of gp61.3, T4L, T4L+gp61.3 mixture, and gp5Lys-gp61.3 complex on a Superdex 75 10/300 GL column.


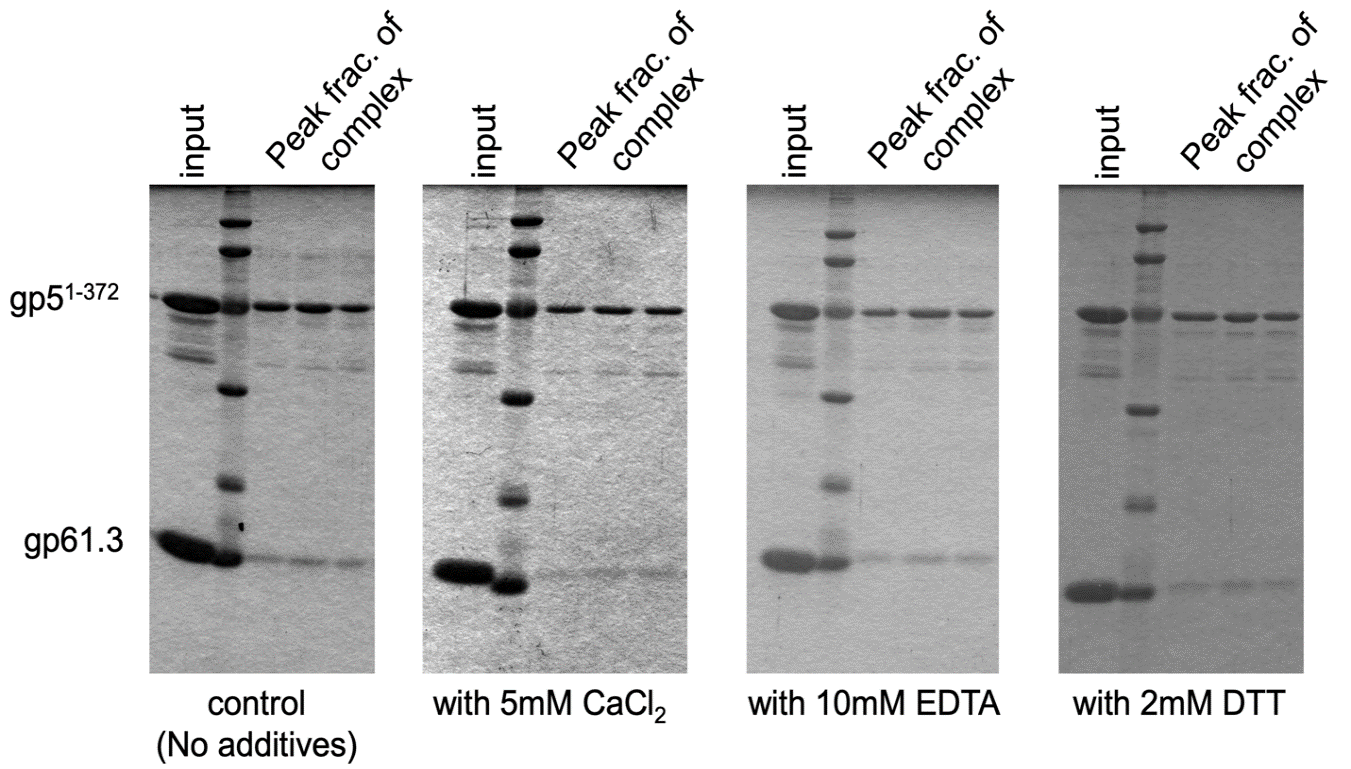


**Figure S3.** Analysis of the gp61.3-gp5 complex formation in the presence of various additives. The composition of the peak fractions of size exclusion chromatographies (the same HiLoad 16/600 Superdex 200 prep grade column) is visualized with the help of SDS-PAGE.


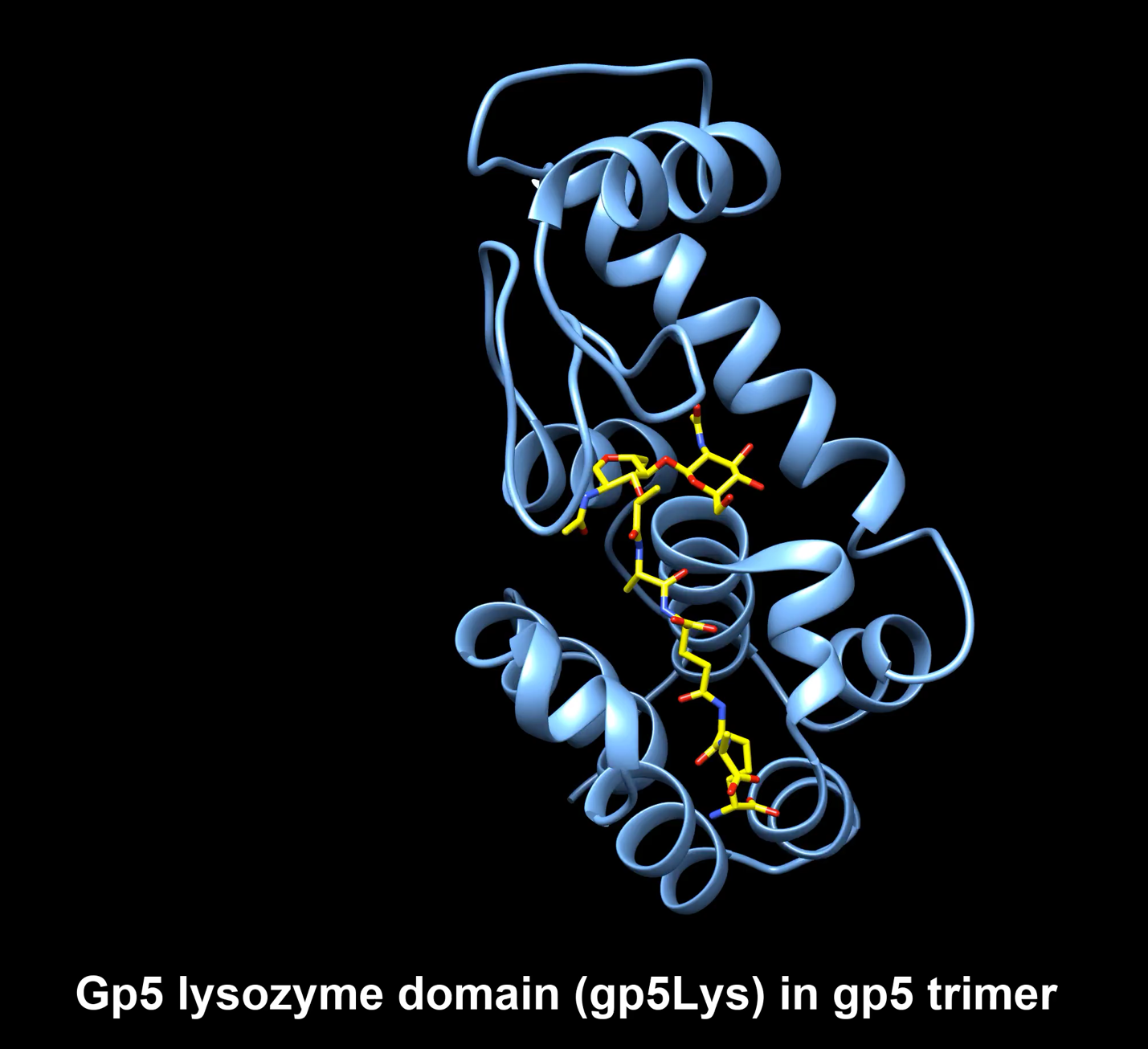


**Video S1.** A comparison of the gp5 lysozyme domain (gp5Lys) conformation in the gp5 trimer with that in complex with gp61.3. Gp5Lys and gp61.3 are colored light blue and orange, respectively. The two structures are superimposed and morphed using UCSF Chimera [36]. The interpolated structures of the morph are colored green. The repeating unit of the *Escherichia coli* peptidoglycan layer is shown as a stick model with carbon atoms colored yellow, oxygen atoms – red, and nitrogen atoms – blue. The location of the peptidoglycan unit is derived from the superposition of T4L with the unit bound to its active site onto the structure of gp5Lys. The T4L is not shown for clarity.
